# Identifying Inhibitors of Inflammation: A Novel High-Throughput MALDI-TOF Screening Assay for Salt-Inducible Kinases (SIKs)

**DOI:** 10.1177/2472555217717473

**Published:** 2017-07-10

**Authors:** Rachel E. Heap, Anthony G. Hope, Lesley-Anne Pearson, Kathleen M. S. E. Reyskens, Stuart P. McElroy, C. James Hastie, David W. Porter, J. Simon C. Arthur, David W. Gray, Matthias Trost

**Affiliations:** 1 MRC Protein Phosphorylation & Ubiquitylation Unit, University of Dundee, Dundee, Scotland, UK; 2 Institute for Cell and Molecular Biosciences, Newcastle University, Newcastle-upon-Tyne, UK; 3 Drug Discovery Unit, University of Dundee, Dundee, Scotland, UK; 4 Division of Cell Signalling and Immunology, University of Dundee, Dundee, Scotland, UK

**Keywords:** MALDI TOF, mass spectrometry, salt inducible kinases, kinase, high-throughput screen, inflammation, drug discovery, macrophage, interleukin-10

## Abstract

Matrix-assisted laser desorption/ionization time-of-flight (MALDI TOF) mass spectrometry has become a promising alternative for high-throughput drug discovery as new instruments offer high speed, flexibility and sensitivity, and the ability to measure physiological substrates label free. Here we developed and applied high-throughput MALDI TOF mass spectrometry to identify inhibitors of the salt-inducible kinase (SIK) family, which are interesting drug targets in the field of inflammatory disease as they control production of the anti-inflammatory cytokine interleukin-10 (IL-10) in macrophages. Using peptide substrates in in vitro kinase assays, we can show that hit identification of the MALDI TOF kinase assay correlates with indirect ADP-Hunter kinase assays. Moreover, we can show that both techniques generate comparable IC_50_ data for a number of hit compounds and known inhibitors of SIK kinases. We further take these inhibitors to a fluorescence-based cellular assay using the SIK activity-dependent translocation of CRTC3 into the nucleus, thereby providing a complete assay pipeline for the identification of SIK kinase inhibitors in vitro and in cells. Our data demonstrate that MALDI TOF mass spectrometry is fully applicable to high-throughput kinase screening, providing label-free data comparable to that of current high-throughput fluorescence assays.

## Introduction

The cells and pathways surrounding the process of inflammation are incredibly complex, which in turn has established a prolific platform for research that has revealed of a wide range of potential new drug targets. This is particularly true in the case of macrophages, which are key players in the innate immune response and exhibit great plasticity in response to various environmental stimuli. Macrophage stimulation results in distinct phenotypic changes ranging from pro- to anti-inflammatory activation states, as well as many in between.^1^ Several chronic inflammatory and autoimmune diseases, such as Crohn’s disease and rheumatoid arthritis, have been implicated in the switch of innate macrophages to a proinflammatory state. These effects can be countered by interleukin-10 (IL-10), a potent repressor of proinflammatory cytokine production.^2,3^ Innovative therapeutic approaches targeting these diseases could exploit this macrophage plasticity in an attempt to reduce the inflammatory response and alleviate patient symptoms.

Current work in this field has highlighted that one or potentially all three isoforms of salt-inducible kinases (SIKs) play a major regulatory role in macrophage phenotype switching by negatively regulating the production of the anti-inflammatory cytokine IL-10.^4–6^ SIKs belong to the serine/threonine protein kinase family and have been implicated in a wide range of biological processes such as insulin signal transduction and metabolic regulation, but their importance in the inflammatory response is best established.^7,8^ Previously, it was shown that prostaglandins activate protein kinase A (PKA), which prevents SIKs from phosphorylating its substrates in cells. Subsequent dephosphorylation of the SIK target CREB-related transcriptional coactivator 3 (CRTC3) at Ser62, Ser162, Ser329, and Ser370 leads to its nuclear translocation promoting CREB-dependent gene transcription and increased IL-10 production.^6,9,10^ Consequently, small-molecule inhibitors affecting SIKs have been shown to increase IL-10 production, thereby switching macrophages to an anti-inflammatory phenotype.^4,11,12^ This suggests that SIKs are attractive drug targets to combat chronic inflammatory diseases.^13^ As kinases are dysregulated in many diseases and are considered excellent drug targets,^14^ methods for kinase profiling and screening are well established. However, current noncellular methods of kinase profiling are often indirect, measuring fluorescence of labeled nonphysiological peptides or adenosine diphosphate (ADP), and/or inclusion of coupling enzymes, or, if direct, require the incorporation of ^33^P through radiolabeled adenosine triphosphate (ATP), which has become unpopular for safety reasons.

A previously very low-throughput technology that could rival these methods in the future is mass spectrometry (MS), which is frequently used to identify and characterize biomolecules. Recent developments based on electrospray ionization (ESI) such as RapidFire^15,16^ or acoustic loading of the mass spectrometer^17^ have substantially improved the throughput in mass spectrometry–based label-free systems, but ESI suffers from the need to clean up samples due to the low tolerance for salts and other compounds. Matrix-assisted laser desorption/ionization (MALDI)^18^ mass spectrometry, on the other hand, is tolerant to a number of standard buffer components^19^ and has rapidly become more popular in the field of drug discovery due to requiring very small sample quantities, minimal sample cleanup, and, most important for high-throughput screening (HTS), very high speed. Historically, MALDI is the most validated and robust surface ionization method whereby the analyte is mixed with a matrix, typically low molecular weight organic acids, and then co-crystalized on a metal target.^20^ Laser irradiation of the dried droplet facilitates proton transfer from the matrix to the analyte, thus ionizing the analyte and allowing MS detection. Designed to be a soft ionization method specifically suitable for quantitatively detecting biomolecules in a single charge state, MALDI is particularly suitable for coupling to time-of-flight mass spectrometers (TOF-MS). MALDI TOF technology has therefore already found traction in the HTS field with its application to label-free screening for inhibitors for deubiquitinating enzymes by measuring the formation of monoubiquitin, an 8.5-kDa protein, as well as for a histone demethylase and an acetylcholinesterase, where it was also shown that this technology can be expanded to an ultra-HTS platform.^21,22^

Here we describe the development and validation of a novel MALDI TOF assay targeted at screening compounds against the kinase SIK2, which has previously been indicated as being an interesting therapeutic target. We compare our MALDI TOF data from a small compound screen to a conventional ADP fluorescence assay, showing good correlation between these methods for the positive hits. Finally, we apply a fluorescence-based assay to further characterize SIK kinase inhibitor candidates in cells.

## Materials and Methods

### Materials

DMSO, α**-**cyano-4-hydroxycinnamic acid (CHCA), trifluoroacetic acid (TFA), magnesium acetate, Tris-HCl, dithiothreitol (DTT), ATP, ammonium citrate dibasic, high-performance liquid chromatography (HPLC)–grade water, HPLC-grade acetonitrile, and HPLC-grade isopropanol were purchased from Sigma-Aldrich (St. Louis, MO). Small-molecule inhibitors staurosporine, HG-9-91-01, MRT199665, MRT67307, KIN112, human full-length SIK2 (expressed in insect cells), and the peptide substrates CHKtide (KKKVSRSGLYRS- PSMPENLNRPR) and NUAK peptide (ALNRTSSDSA- LHRRR) were provided or produced in-house and are available to request from our reagent’s website (https://mrcppureagents.dundee.ac.uk/). 1536 MTP AnchorChip targets and peptide calibration standard II were purchased from Bruker Daltonics (Bremen, Germany).

### Preparation of Plates for Screening

For single-concentration screening, 15 nL (MALDI TOF assay) or 24 nL (ADP Hunter assay; DiscoverX, Fremont, CA, USA, UK) of a 10-mM compound solution in DMSO was transferred into 384-well HiBase, nonbinding microtiter plates (Greiner, Stonehouse, UK), using an Echo 550 acoustic dispenser (Labcyte, San Jose, CA, USA), to give a final screening concentration of 30 µM. Staurosporine at 10 µM was used as a positive control in column 24, with column 23 containing only DMSO as the negative control. The Dundee Drug Discovery Unit (DDU) small-molecule kinase library contains 2648 commercially available ATP mimetics describing a diverse region of kinase pharmacophore space.

### SIK ADP Hunter Assay

The ADP Hunter technology (DiscoverX) is a coupled enzyme assay that detects the production of the resorufin fluorophore as a downstream consequence of the conversion of ATP to ADP. It therefore provides a generic high-throughput assay for the measurement of kinase enzyme activity. Screening of full-length GST-tagged human SIK2 was performed using CHKtide as a substrate peptide. Compounds were stamped into 384-well, low-volume, black-wall, assay plates (Greiner Bio-one, Stonehouse,UK) as described above. The assay window was defined by addition of DMSO in the presence (no effect) or absence of enzyme (maximum effect) to defined control wells. Using a Wellmate liquid dispenser (Thermo Scientific, Hemel-Hempstead, UK), 4 µL of a 2× enzyme solution (ADP Hunter Plus assay buffer, 1 mM DTT, enzyme) was dispensed into all wells and preincubated for 20 min at room temperature. The assay enzyme reaction was initiated by the addition of 4 µL of a 2× substrate solution (ADP Hunter Plus assay buffer, 100 µM CHKtide, 200 µM ATP Gold) such that the final assay concentration of SIK2 was 10 nM. Assay plates were incubated for 30 min at room temperature, after which the reaction was stopped by the addition of 4 µL ADP Hunter Plus Reagent A and 8 µL ADP Hunter Plus Reagent B. Following a further incubation for 30 min at room temperature, resorufin-elicited fluorescence was measured (ex 550 nm; em 595 nm) using a PHERAstar FS (BMG Labtech, Aylesbury, UK).

### SIK MALDI TOF Assay

In this assay, the appearance of phosphorylated CHKtide product at *m/z* 2779.47 was monitored with respect to the CHKtide substrate at *m/z* 2700.47 using MALDI TOF mass spectrometry.

Screening assays were performed with an XRD-384 Automated Reagent Dispenser (Fluid, Nether Alderley, UK) by dispensing 3 µL of freshly prepared assay buffer (50 mM Tris/HCl, 10 mM magnesium acetate, 2 mM DTT, pH 7.5) and 1 µL of 15 nM SIK2 prepared in assay buffer onto prepared compound plates followed by a 30-min incubation at 30 °C. The reaction was then initiated by addition of 1 µL substrate solution containing a mixture of CHKtide and ATP to a final concentration of 1 µM and 100 µM, respectively. Plates were then returned to incubation at 30 °C for 30 min before being quenched by addition of 1.2 µL of TFA to a final concentration of 2%.

### MALDI MS Target Spotting

First, 9.04 mg dibasic ammonium citrate was dissolved in 1 mL HPLC-grade water and vortexed to create a 40-mM solution. Then, 10 mg CHCA was weighed out and prepared to a final volume of 1 mL in 50% HPLC-grade ACN and 50% water (v/v), 0.1% TFA, and 10 mM dibasic ammonium citrate and was prepared and placed on a shaker for 30 min to aid dissolution before use.

Quenched samples and freshly prepared CHCA matrix solution were mixed in a 1:1 ratio (v/v) with a Mosquito nanoliter dispenser in an LVSD plate (TTP Labtech, Melbourn, Hertfordshire, UK) and spotted with the dried droplet technique in 200-nL aliquots onto 1536-MTP AnchorChip targets (Bruker Daltonics). Each sample was spotted in duplicate and the spotted targets allowed to air dry before MALDI-MS analysis.

MTP AnchorChip targets were cleaned before each use by sonication in HPLC-grade isopropanol for 2 min, followed by sonication in 30% acetonitrile and 0.1% TFA for 2 min. Targets were then placed in a clean room and allowed to ambient dry before use.

### MALDI-MS Analysis

A RapifleX MALDI TOF/TOF mass spectrometer (Bruker Daltonics) equipped with a Smartbeam 3D laser was used in positive ion mode with Compass 2.0 control for all data acquisition. Samples were run in automatic mode (AutoXecute; Bruker Daltonics), acquiring 5000 shots at a 10-kHz frequency per spot in a random walk on spot laser ablation pattern and M5 Smartbeam Parameter at a 25-µm × 25-µm scan range. Ionization was achieved using a fixed laser power of 70% (laser attenuator offset 7%, range 30%) and detected by the FlashDetector, Bruker, Bremen, Germany at a detector gain of ×2 in the 2500 to 2800 *m/z* mass range. Samples were analyzed in Reflector mode with optimized voltages for reflector 1 (20.82 kV), reflector 2 (1.085 kV), and reflector 3 (8.8 kV), ion sources (ion source 1, 20 kV, PIE 2.66 kV), lens (11.3 kV), and a pulsed ion extraction of 200 ns. A novel 10-bit digitizer was used at a sampling rate of 5.00 GS/s.

Spectra were accumulated with FlexControl software (v4.0) and processed using FlexAnalysis software (v4.0) (Bruker Daltonics). Peaks were centroid detected with a signal-to-noise threshold of 6.00 to detect only the intense peaks before being processed with a Savitzky-Golay smoothing algorithm (0.05 *m/z* width, one cycle) and “TopHat” baseline subtraction. External calibration was performed before each new target in Cubic Enhanced mode with Pepmix II calibrant (Bruker Daltronics) containing seven peptides: angiotensin II [M+H]^+^ = 1046.54, angiotensin I [M+H]^+^ = 1296.68, substance P [M+H]^+^ = 1347.74, bombesin [M+H]^+^ = 1619.82, ACTH clip (1–17) [M+H]^+^ = 2093.09, ACTH clip (18–39) [M+H]^+^ = 2465.20, and somatostatin (28) [M+H]^+^ = 3147.47. Internal calibration was performed using the CHKtide peptide substrate monoisotopic [M+H]^+^ = 2700.60 Th.

### Data Analysis

For enzyme characterization, initial rates in both technologies were determined using time-course experiments under conditions of either excess ATP or CHKtide. The rates were plotted against variable substrate concentration and subjected to standard Michaelis-Menten analysis to derive K_m_ and V_max_ values. Values were normalized for volume and protein concentration across the two technologies for comparative purposes.

MALDI TOF data processed by the FlexAnalysis 4.0 software were exported as a .csv file using FlexAnalysis Batch Process (Compass 2.0) and further processed in Microsoft Excel (Microsoft, Redmond, WA). For peak area calculation, the isotopic distribution was taken into account by determining a peak area from 2699.4 to 2702.6 *m/z* for the unphosphorylated CHKtide and 2779.8 to 2782.2 *m/z* for the phosphorylated CHKtide.

SIK2 activity was measured by the percent phosphorylation (%P) of the CHKtide substrate by the following equation:

$$(\frac{\left(\mathrm{Peak Area Phosphorylated Product}\right)}{\left(\mathrm{Peak Area Phos product})+(\mathrm{Peak Area Substrate}\right)})\times 100.$$

Raw data from the ADP Hunter assay (relative fluorescence units [RFU]) were also exported as a comma delimited (.csv) file. All subsequent primary single-point screening data processing and analysis for both assays were conducted within Activity Base version 8.1.2.12 using Activity Base XE Runner version 8.1.1 (IDBS, Guilford, UK). Data were normalized and expressed as a percentage effect (PE) value for each test compound as follows:

MALDI TOF data:

$$\mathrm{PE}=(\frac{\%P_{\mathrm{test}}-\%P_{\mathrm{high}}}{\%P_{\mathrm{low}}-\%P_{\mathrm{high}}}\times 100)$$

ADP Hunter data:

$$\mathrm{PE}=(\frac{\mathrm{RF}U_{\mathrm{test}}-\mathrm{RF}U_{\mathrm{high}}}{\mathrm{RF}U_{\mathrm{low}}-\mathrm{RF}U_{\mathrm{high}}}\times 100),$$

where test is the signal associated with the test compound, high is the median of the no-effect signal (reaction in presence of enzyme only), and low is the median of maximum effect control signal (reaction in absence of enzyme [biochemical] or presence of staurosporine [MALDI TOF]).

The performance of the assay on each screening plate was evaluated using internal controls to determine robust signal-to-background (s:b) and robust Z′ values, which were calculated as follows:

$$s:b=\frac{\mathrm{high}}{\mathrm{low}},$$

$$Z\prime =1-(\frac{\left(3\times \mathrm{sMADhigh})+(3\times \mathrm{sMADlow}\right)}{\left(\mathrm{high}-low\right)}),$$

where high refers to either RFUhigh or %Phigh and low corresponds to either RFUlow or %Plow, as appropriate. sMADhigh is 1.4826 × median absolute deviation of signal associated with the no-effect control, and sMADlow is the 1.4826 × median absolute deviation of signal calculated for the maximum effect control.

All database querying for global data analysis and report creation was undertaken using the Dotmatics Browser (version 4.9.623.45008-s) and Vortex analysis software (version 2015.12.46651.25; Dotmatics, Bishop Stortford, UK).

### SIK Cellular Assay

The human bone osteosarcoma cell line U2OS (ATCC HTB-96) was subcultured and grown in 96-well plates (µClear bottom, cat. 655090; Greiner, Stonehouse, UK). After 24 h, cells were treated for 1 h with the small-molecule inhibitors staurosporine, HG-9-91-01, KIN112, MRT67307, and MRT199665. DMSO at 0.1% and secondary antibody-only controls were included on each plate. All compounds started at 10 µM and were serially diluted with eight replicates per treatment group. Cells were fixed in 4% paraformaldehyde in phosphate-buffered saline (PBS) and permeabilized with 1% NP-40 in PBS for 10 min each, respectively. Following a blocking step with 3% goat serum (Sigma) for 45 min, cells were incubated overnight at 4 °C with rabbit anti-CRTC3 (1:350; Abcam, Cambridge, UK) followed by 1 h with goat anti-rabbit Alexa 488 (1:300; Invitrogen, Carlsbad, CA) and 1 µg/mL 4′,6-diamidino-2-phenylindole (DAPI; Invitrogen). Blocking and antibody solutions were made in 1% bovine serum albumin (BSA)–PBS. Plates were subsequently imaged on a PerkinElmer (Coventry, UK) Operetta high-content wide-field fluorescence imaging system with a 20× objective and identical parameters used for all well and plates: DAPI (em 445 nm), Alexa 488 (em 525 nm), and digital phase contrast (ex 740 nm) with four fields of view imaged per well in an identical pattern. Quantification was performed using the Columbus database and analysis software. Nuclear intensity of CRTC3 was quantified via nuclear identification with DAPI, with subsequent subtraction of the secondary-only control (Alexa 488 signal) from all samples. Results are expressed as mean fluorescence intensity ± standard deviation (SD).

## Results and Discussion

**Figure 1.**
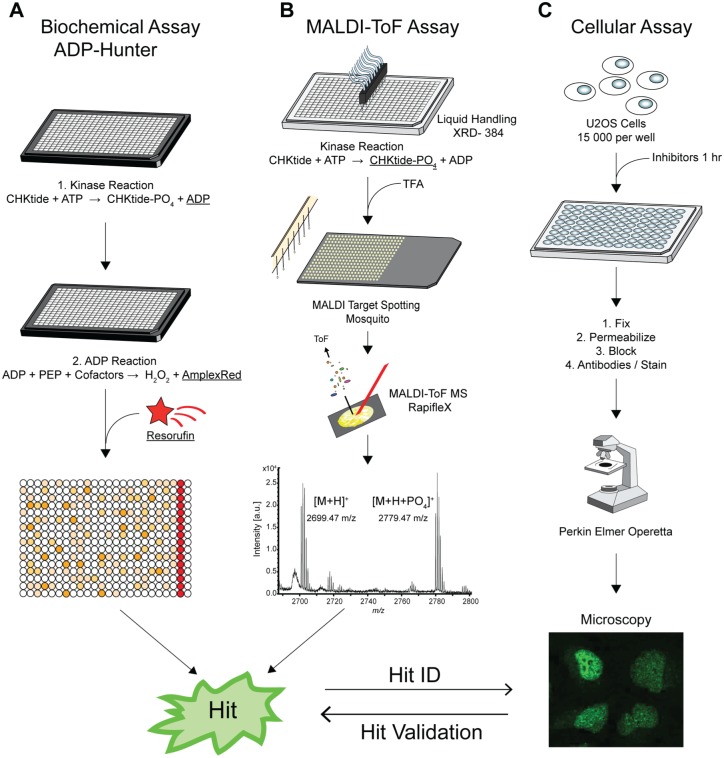
Workflows to identify salt-inducible kinase (SIK) kinase inhibitors. (**A**) The biochemical kinase assay was performed using ADP Hunter, which uses an enzyme-coupled reaction that produces a red-shifted fluorescence signal that is directly proportional to the amount of adenosine diphosphate (ADP) produced by the kinase reaction. (**B**) For the matrix-assisted laser desorption/ionization time-of-flight (MALDI TOF) assay, enzyme reactions were performed in 384-well format with an XRD liquid handler. Samples were spotted onto 1536-anchorChip targets using a Mosquito liquid handling robot and analyzed on a Rapiflex mass spectrometer. (**C**) For the fluorescence assay, 15000 U2OS cells per well were treated with inhibitors for 1 h in a 96-well format. Cell were fixed, permeabilized, blocked, and incubated with DAPI and an antibody against CRTC3. CRTC3 translocation was analyzed on a PerkinElmer Operetta system. ATP, adenosine triphosphate; MALDI TOF, matrix-assisted laser desorption/ionization time-of-flight.

To screen for inhibitors of the SIK2 kinase, we developed and applied three distinct high-throughput assays (**Fig. 1**): a well-established fluorescence-based, enzymatically coupled assay, ADP Hunter; a label-free MALDI TOF assay screening for the phosphorylated peptide substrate; and a cell-based assay using the nuclear translocation to the SIK substrate CRTC3 in response to SIK inhibition. For the in vitro kinase assays, human, recombinant full-length SIK2 was used. We chose two readily available synthetic peptides as substrates for SIKs: NUAK peptide, which is derived from NUAK2, a stress-activated kinase from the LKB1 signaling pathway,^23^ and CHKtide, a peptide derived from the CHK1 protein kinase involved in the DNA repair pathway, which was recently identified in a kinase screen to be a good SIK substrate (unpublished data).

### Development of a MALDI-TOF Assay for SIK2 Kinase

First, we optimized MALDI TOF assay conditions for analyzing a peptide substrate. The choice of matrix and spotting technique often has strong effects on the MALDI-MS analysis, and there are many matrices commercially available. Optimization for these specific peptides showed best results when spotted with CHCA in 50% acetonitrile, 0.1% TFA, and spotting 200-nL sample by a dried droplet method^23^ using a Mosquito nanoliter dispenser, which allowed uniform deposition onto the target and matrix. The total time taken to spot a full 1536 target was ~30 min, which is comparable to other MALDI TOF assay spotting times.^22^

**Figure 2.**
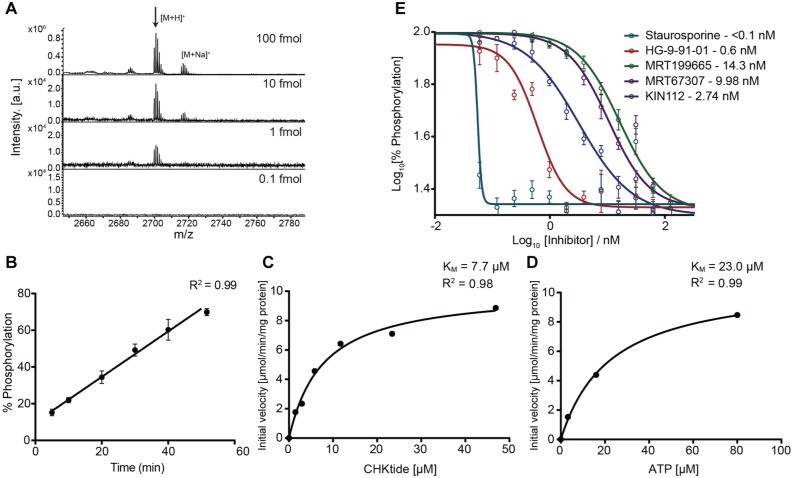
Matrix-assisted laser desorption/ionization time-of-flight (MALDI TOF) salt-inducible kinase (SIK) kinase assay specifications. (**A**) The MALDI TOF spectra of CHKtide in a dilution series. The assay is very sensitive, with 1 fmol still showing high signal to noise. (**B**) Linearity of MALDI TOF SIK kinase assay. The assay is linear between 10 and 50 min and over a peptide occupancy of ~15% to 75%. (**C**, **D**) Enzymatic constants determined by MALDI TOF mass spectrometry for CHKtide (**C**) and adenosine triphosphate (**D**) for SIK2. (**E**) IC_50_ dose-response curves for five inhibitors against SIK2 as determined by the MALDI TOF assay.

We next tested which of the two substrates performed best. The majority of peptides ionize well by MALDI, but it has been shown in the past that ionization efficiency is dependent on the gas-phase basicity of peptides.^24,25^ Thus, arginine- and lysine-rich peptides tend to have better responses, and therefore we expected that the CHKtide substrate would perform better as it contains more basic residues that are also more distributed over the whole peptide sequence. Both peptides were tested for sensitivity, with CHKtide indeed proving to perform better with a detection limit of 1 fmol on target with good signal to noise (**Fig. 2A**) compared to the NUAK peptide (**Suppl. Fig. S1**), which was also detectable at 1 fmol but exhibited a sevenfold lower signal to noise. Both peptides formed only minimal salt adducts and oxidized species; therefore, CHKtide was chosen to be the substrate of choice for this assay.

With the peptide substrate and spotting technique established, the enzymatic reaction was assessed for detection of the phosphorylated product, as well as the reaction kinetics. Many buffers of biochemical assays contain components that are not considered “MS-friendly” as they suppress ionization of the analytes. MALDI TOF has a low tolerance for some salts such as NaCl, as it often leads to adduct formation, thus making interpretation and quantification of peptide spectra difficult.^26^ We therefore modified established kinase assay buffers to be more compatible with MALDI TOF, guided by a recent investigation into MALDI-MS–friendly buffers.^19^ Initial experiments in the ADP Hunter assay showed that the removal of NaCl from the assay buffer did reduce the signal window but did not alter the rank order of inhibitors. We therefore removed NaCl from the assay buffer of the MALDI assay. Final assay conditions gave a robust and reproducible response of both the phosphorylated and unphosphorylated peptide, as well as being comparable with the conventional ADP Hunter assay that is performed in-house.

Using the MALDI TOF assay, we determined that the phosphorylation of CHKtide through SIK2 (**Fig. 2B**) was linear up to 60 min between ~15% and 75% occupancy. From these data, an optimal point of 30 min was chosen to quench the reaction, thus allowing a conversion of approximately 50% of the substrate peptide to its phosphorylated equivalent. When comparing ADP Hunter and MALDI TOF assay enzyme concentrations, similar final enzyme concentrations were used. However, the MALDI TOF assay uses fourfold less enzyme due to the reduction in assay volume. Equally, peptide substrate consumption of the MALDI assay is up to 300 times lower in concentration and 10 times lower in volume, mostly due to the high sensitivity of the mass spectrometer.

We further characterized enzymatic constants for CHKtide (**Fig. 2C**) and ATP (**Fig. 2D**) using the MALDI-TOF assay. Both the CHKtide and ATP K_m_ constants were very similar for SIK2 and comparable to the K_m_ constants determined in the ADP Hunter assay.

Furthermore, we determined for SIK2 a 15-point dose-response curves for five known inhibitors (**Fig. 2E**). Staurosporine is a well-known, broad, kinase inhibitor with a high potency, whereas HG-9-91-01, MRT199665, MRT67307, and KIN112 are all previously described as targeting SIKs at a nanomolar concentration, with HG-9-91-01 being the most potent of the four.^4^ These inhibitors were chosen as all five are ATP competitive, which reflects the small molecular library of ATP mimetics that was selected for the screen. Overall, the IC_50_ values were all in the low nanomolar range and about 0.5 to 1 order of magnitude higher potency than published biochemical assays,^4^ suggesting a specific trend in the MS-based assay (**Suppl. Fig. S2** and **Suppl. Table S2**). However, this is not unexpected as previous IC_50_ values were determined by less direct measurements, such as IL-10 messenger RNA (mRNA) induction or cell-based methods. In cells, a higher ATP concentration and the presence of other potential targets such as AMPKs and a number of protein tyrosine kinases, cellular penetration of the compound, and inclusion of high concentrations of proteins that may bind to compound could contribute to differences seen for the inhibitor potencies. Staurosporine, which serves as a control, appears significantly more potent than the four other control inhibitors (**Suppl. Fig. S2** and **Fig. 2E**), and we observe a poorer slope fit, thus suggesting that other factors such as tight-binding could be causing an overestimation in the potency of staurosporine. This could be a result of the IC_50_ value being near or below the SIK2 concentration in the assay, which would produce a tight-binding condition and therefore underrepresent the true potency of the inhibitor. Comparison of the four other control inhibitors leads us to believe that the ADP Hunter and MALDI-TOF assay have similar biochemical pharmacology.

### MALDI TOF SIK2 Assay Is Comparable to an ADP Hunter Assay

Next, we screened the DDU small-molecule kinase library against SIK2 using both the MALDI TOF assay and the ADP Hunter assay. This library contains 2648 commercially available ATP mimetics describing a diverse region of kinase pharmacophore space, consisting of nine 384-well assay plates. Both assays were demonstrated to be robust methods for screening, with calculated mean robust Z′ and robust s:b values (**Table 1**).

**Table 1.** Robustness of SIK2 Assays.

SIK2 Assay	Robust Z′	Robust s:b
MALDI TOF	0.5	3.0
ADP Hunter	0.9	2.8

MALDI TOF, matrix-assisted laser desorption/ionization time-of-flight; s:b, signal to background; SIK2, salt-inducible kinase 2.

**Figure 3.**
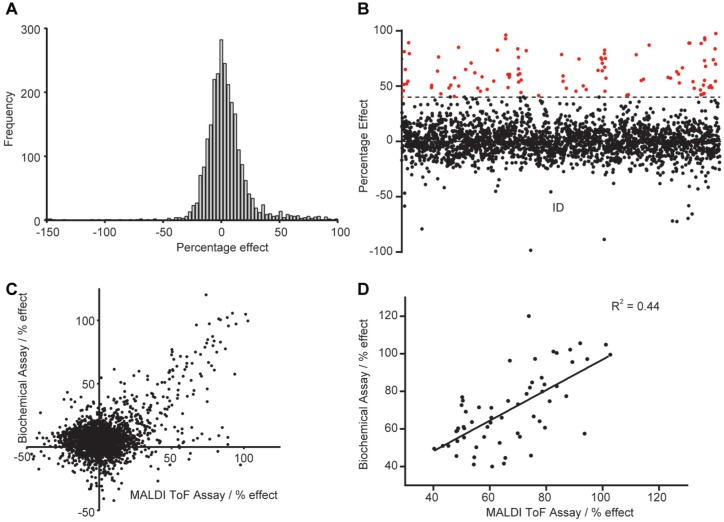
Comparison between biochemical and matrix-assisted laser desorption/ionization time-of-flight (MALDI TOF) salt-inducible kinase 2 (SIK2) assay. (**A**) Blot showing significant hits (>40% inhibition, in red) of the MALDI TOF assay. Data also show some apparent activators (<–50% effect). (**B**) Data distribution of the MALDI TOF SIK2 kinase assay shows a normal distribution. (**C**) Comparison of percentage inhibition for all compounds tested between the biochemical ADP Hunter assay and the MALDI TOF assay. (**D**) Comparison of “hits” (>40% inhibition) from both biochemical ADP Hunter assay and MALDI TOF SIK2 assay showing high correlation between the two assays.

The output of both screens for SIK2, expressed as percentage effect for MALDI TOF (**Fig. 3A**) and ADP Hunter (**Suppl. Fig. S3**), exhibited a normal distribution, with calculated mean percentage effects of 2.3 (MALDI TOF) and 7.6 (ADP Hunter) for SIK2. Standard deviations of the whole screening data set were comparable with 20.6% (MALDI TOF) and 14.6% (ADP Hunter) effect. For both screens, compounds producing a statistically significant effect were defined as those producing a percentage effect of greater than or equal to mean effect ± 3 SD of the whole screening data set. In addition, the number of compounds producing percentage effect greater than 40% (**Fig. 3B** and **Suppl. Fig. S4**) was also determined for each assay. A summary of the number of hit compounds is summarized in **Table 2**.

**Table 2.** Number of Compound Hits.

SIK2 Assay	PE ≥ Mean ± 3 SD	PE ≥40%	Unique Hits: PE ≥ Mean ± 3 SD	Unique Hits: PE ≥40%
MALDI TOF	45	107	14	45
ADP Hunter	59	79	26	16

MALDI TOF, matrix-assisted laser desorption/ionization time-of-flight; PE, percentage effect; SIK2, salt-inducible kinase.

As expected for single-shot screens, the number of hits was not identical for the two screens, but they did correlate (*R*^2^ = 0.44) between the percentage effects measured for compounds producing greater than 40% effect in the two assays (**Fig. 3D**). With further replicates, we would expect a tighter correlation between the hits of the two assays. Eighty percent of the ADP Hunter assay hits (PE >40%; 59 compounds) were identified in both screens (28 with PE >3 SD). There are also 16 hits that were only identified in the ADP Hunter assay and a significant number of hits (45) that were only present in the MALDI TOF assay (**Fig. 3C**), suggesting that direct analysis of the peptide product might produce additional hits compared to the indirect ADP assay. Moreover, nine compounds appeared to increase the kinase reaction rate that were not identified in the ADP Hunter assay. These hits appeared to be genuinely increasing the product formation (**Suppl. Fig. S5**), and more future work will be needed to characterize the differences between the ADP Hunter and the MALDI TOF assays.

### Cellular SIK Inhibition Screen

To take these compounds further and screen SIK kinase activity in cells, we developed a fluorescence-based assay that was built on the fact that CRTC3, a nuclear coactivator for the transcription factor CREB, translocates into the nucleus when SIK kinases are inactivated. This is driven by dephosphorylation of four serines that are targeted by SIKs. This nuclear translocation is essential for activating the transcriptional program for IL-10 production and polarization toward regulatory-like, anti-inflammatory macrophages.^4,6,26^

**Figure 4.**
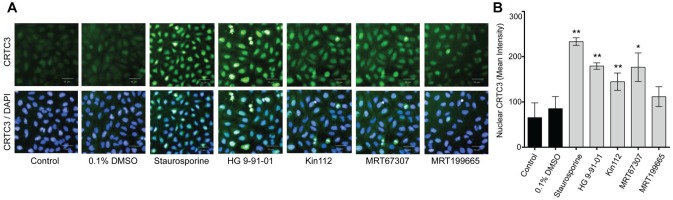
Nuclear localization of CRTC3 with small-molecule salt-inducible kinase (SIK) inhibitors. U2OS cells were treated for 1 h with 0.1% DMSO or small-molecule inhibitors serially diluted. (**A**) Representative microscopic images at 20× magnification from the high-throughput system at 10 µM inhibitor with CRTC3 (Alexa 488, green) and nucleus (DAPI, blue) shown. Scale bar = 50 µm. (**B**) Inhibition of SIK kinases induces strong nuclear translocation of CRTC3. Mean fluorescent intensity of nuclear CRTC3 calculated with data represented as mean ± SD. The *p* values versus 0.1% DMSO are as follows: staurosporine, *p* = 1.31 × 10^−19^; HG 9-91-01, *p* = 4.45 × 10^−14^; Kin112, *p* = 2.95 × 10^−6^; MRT67307, *p* = 0.0061; MRT199665, *p* = 0.533. *p < 0.01, **p < 0.001 vs 0.1% DMSO.

Human U2OS cells were treated for 1 h with inhibitors, cells were fixed, and nuclear CRTC3 translocation was analyzed by fluorescence-labeled antibody staining on a high-throughput fluorescence imaging platform (PerkinElmer Operetta). The cellular assay showed that nuclear translocation of CRTC3 is enhanced by SIK inhibition. Comparable with our in vitro data, strength of CRTC3 translocation is correlated with the potency of the inhibitors. Staurosporine and HG 9-91-01 are the most potent of the inhibitors compared to DMSO, showing a very dense nuclear localization of CRTC3 (**Fig. 4A**). Kin112 is not as potent, and although MRT67307 does induce localization, this is with much greater variation versus the other compounds. MRT199665 is the least potent of all the inhibitors tested (**Fig. 4B**). These data show that this is a useful platform to differentiate screening hits efficacy for SIK inhibitors at the cellular level.

In this article, we compared the output of MALDI TOF mass spectrometry to the more established ADP hit Hunter technology using SIK enzymes as model kinases and presented a robust cell-based assay providing a suitable downstream compound profiling assay for SIK inhibitors. Both the MALDI TOF and ADP Hunter techniques generated robust assays suitable for both single-point hit discovery and compound profiling. The basic enzyme properties characterized were largely similar as were the hit molecules discovered. In addition, the potency of standard inhibitors was closely aligned. These data extend the target families where MALDI TOF has been demonstrated to be a suitable platform for drug discovery. While MALDI TOF has a higher capital entry cost than the more traditional biochemical methods, it has advantages in terms of higher sensitivity, leading to a reduction in quantities required of both enzyme and substrate. In addition, although not an issue with the chemotypes used in this study, we anticipate that high-throughput MALDI TOF will show less false positives and false negatives from compound interference such as autofluorescence or usage of fluorescent tags on substrates. Moreover, secondary assays such as ADP Hunter might be affected by the compounds themselves. Together, the suite of assays and validation described here established a platform for early drug discovery for the SIK kinases.
